# Herpesvirus Infections in KIR2DL2-Positive Multiple Sclerosis Patients: Mechanisms Triggering Autoimmunity

**DOI:** 10.3390/microorganisms10030494

**Published:** 2022-02-23

**Authors:** Daria Bortolotti, Valentina Gentili, Alessandra Bortoluzzi, Marcello Govoni, Giovanna Schiuma, Silvia Beltrami, Sabrina Rizzo, Eleonora Baldi, Elisabetta Caselli, Maura Pugliatti, Massimiliano Castellazzi, Mercedes Fernández, Enrico Fainardi, Roberta Rizzo

**Affiliations:** 1 Department of Chemical, Pharmaceutical and Agricultural Science, University of Ferrara, 44121 Ferrara, Italy; daria.bortolotti@unife.it (D.B.); valentina.gentili@unife.it (V.G.); giovanna.schiuma@unife.it (G.S.); silvia.beltrami@unife.it (S.B.); sabrina.rizzo@unife.it (S.R.); elisabetta.caselli@unife.it (E.C.); mercedes.fernandez@unife.it (M.F.); 2 Rheumatology Unit, Department of Medical Sciences, University of Ferrara and Azienda Ospedaliero-Universitaria S. Anna, 44124 Ferrara, Italy; alessandra.bortoluzzi@unife.it (A.B.); marcello.govoni@unife.it (M.G.); 3 Division of Neurology, Department of Neuroscience and Rehabilitation, “Sant’Anna” University-Hospital, 44124 Ferrara, Italy; eleonora.baldi@gmail.com; 4 Department of Biomedical and Specialist Surgical Sciences, University of Ferrara, 44121 Ferrara, Italy; maura.pugliatti@unife.it (M.P.); massimiliano.castellazzi@unife.it (M.C.); 5 Interdepartmental Research Center for the Study of Multiple Sclerosis and Inflammatory and Degenerative Diseases of the Nervous System, University of Ferrara, 44124 Ferrara, Italy; 6 Department of Experimental and Clinical Biomedical Sciences, University of Florence, 50121 Florence, Italy; enrico.fainardi@unifi.it

**Keywords:** herpesvirus, multiple sclerosis, NK cell, KIR

## Abstract

In multiple sclerosis (MS), there is a possible relationship with viral infection, evidenced by clinical evidence of an implication of infectious events with disease onset and/or relapse. The aim of this research is to study how human herpesvirus (HHVs) infections might dysregulate the innate immune system and impact autoimmune responses in MS. We analyzed 100 MS relapsing remitting patients, in the remission phase, 100 healthy controls and 100 subjects with other inflammatory neurological diseases (OIND) (neuro-lupus) for their immune response to HHV infection. We evaluated NK cell response, levels of HHVs DNA, IgG and pro- and anti-inflammatory cytokines. The results demonstrated that the presence of KIR2DL2 expression on NK cells increased the susceptibility of MS patients to HHV infections. We showed an increased susceptibility mainly to EBV and HHV-6 infections in MS patients carrying the KIR2DL2 receptor and HLA-C1 ligand. The highest HHV-6 viral load was observed in MS patients, with an increased percentage of subjects positive for IgG against HHV-6 in KIR2DL2-positive MS and OIND subjects compared to controls. MS and OIND patients showed the highest levels of IL-8, IL-12p70, IL-10 and TNF-alpha in comparison with control subjects. Interestingly, MS and OIND patients showed similar levels of IL-8, while MS patients presented higher IL-12p70, TNF-alpha and IL-10 levels in comparison with OIND patients. We can hypothesize that HHVs’ reactivation, by inducing immune activation via also molecular mimicry, may have the ability to induce autoimmunity and cause tissue damage and consequent MS lesion development.

## 1. Introduction

In multiple sclerosis (MS), there is a possible relationship with viral infection, evidenced by clinical evidence of an implication of infectious events with disease onset and/or relapse. The viral infection can directly infect the central nervous system (CNS) and induce an inflammatory response that might result in brain damage. Neurotrophic herpesviruses can enter the CNS evading the host protective immune response, inducing acute cell dysfunction. Subsequently, the latent infection typical of herpesviruses might reactivate resulting in disease relapse. The reactivation of viral infection can induce lymphocyte activation and the secretion of pro-inflammatory cytokines that affect cell-specific functions and enhance neurodegeneration [1]. Disease-associated genetic variants can exacerbate the disease onset [2]. Herpesviridae have shown a possible association with MS, resulting as valuable biomarker candidates of disease progression and therapy outcome. Their preferential tropism for CNS and their latent persisting infections that enable their immune-escape, account for their potential pathogenic role in neuroinflammation, resulting in the persistence of chronic inflammation and the accumulation of neurological deficits. Epstein–Barr virus (EBV) and human herpesvirus 6 (HHV-6) have been identified in pathological and sero-epidemiological studies. EBV reactivation has been linked to disease activity in early MS suggesting a possible implication in MS immunopathology [3]. HHV-6 active infection seems to be involved in MS exacerbations [4], and reactivation may have a role in triggering autoimmune response and tissue damage associated with MS lesion development [5,6]. Our group showed that HHV-6 supports a productive, low level of replication in the CNS of patients at the early stages of the disease [7].

Recent observations suggest that innate immunity, and in particular natural killer (NK) cells, might be involved in the etiology of MS [8]. NK cells’ regulation is controlled by NK membrane receptors: leukocyte immunoglobulin-like receptors (LILR), C-type lectin, killer immunoglobulin-like receptors (KIR), natural cytotoxicity receptors (NCRs) and CD2. KIRs are transmembrane glycoproteins encoded by 15 highly polymorphic genes, with structural domains that characterize their functions [9]. The KIRs have different numbers of the extracellular domains (2D and 3D), and a cytoplasmic tail with a different length (L (long) or S (short)) that determines protein function (inhibitory or activating, respectively) [10], with KIR2DL4 as the unique long-tailed activating KIR. The association of KIR gene polymorphisms has already been demonstrated in several autoimmune disorders such as rheumatoid arthritis, ankylosing spondylitis and inflammatory bowel disease [11,12,13]. In particular, KIR receptors might have a protective or detrimental role in autoimmune diseases due to their inhibitory or activating control of NK cells, the functions of which can be suppressed or enhanced in predisposed subjects.

We have recently showed that MS patients characterized by the expression of KIR2DL2 on NK cell surface are more susceptible to HHV infection [14,15,16]. In particular, NK cells from MS patients expressing KIR2DL2 are less activated towards HHV infection. On the contrary, control KIR2DL2-positive and negative subjects presented no statistical differences in the activation status. Interestingly, other authors have observed a correlation between KIR2DL2 and the HHV infection reactivation in healthy subjects [17]. The authors recognized receptor/ligand pair KIR2DL2/HLA-C1 as a predisposing factor for HSV-1 infection and reactivation. These results suggest a possible role of KIR2DL2 in HHV infection susceptibility in the human population, that affects NK cell activation only in MS patients. We can hypothesize that HHVs’ reactivation, by inducing immune activation via molecular mimicry, may have the ability to induce autoimmunity and cause tissue damage and consequent MS lesion development.

The aim of this research is to study how such dysregulation of the innate immune system could impact autoimmune responses in MS.

## 2. Materials and Methods

### 2.1. Subjects

In total, 100 MS relapsing remitting patients, in remission phase (mean age: 39 ± 10 years), recruited at the MS Centre of the Department of Neurology, University of Ferrara, Italy, from 2015 to 2018 (Supplementary Table S1a), 100 healthy controls (mean age: 38 ± 11 years) and 100 subjects with other inflammatory neurological diseases (OIND) (mean age: 37 ± 12 years) (NLES: neuro-lupus) (Supplementary Table S1b) were recruited. The samples were collected after informed consent, following the acceptance by the Area Vasta Emilia Centro (N: 01052016). MS was defined on the basis of McDonald’s classification [18]. The patients had relapsing remitting MS according to Lublin [19]. Disease classification was assessed during sample collection using Kurtzke’s Expanded Disability Status Scale (EDSS) [20] (mean value at entry: 2.0 ± 1.0, range from 0.0 to 5.6) (Supplementary Table S1a). At entry, the patients had no signs of acute infections, fever or other symptoms. The patients were not receiving potential disease-modifying therapies (e.g., methylprednisolone or azathioprine, glatiramer acetate or interferon-beta) during the 6 months preceding the study. OIND patients satisfying the 1997 revised American College of Rheumatology Criteria regularly attending the Lupus Clinic of the Rheumatology Unit, Department of Medical Sciences, Sant’Anna Hospital, University of Ferrara, Italy were recruited during the same period [21]. Neuropsychiatric manifestations were assessed in accordance with the 1999 ACR nomenclature and case definitions and diagnoses followed the EULAR recommendations [22]. The attribution of NP events was based on physician judgement and considering ACR ‘association’ and ‘exclusion’ factors (i.e., their absence favors attribution to SLE), and as ‘SLE-favoring factors’ of the Italian Study Group on the NPSLE validated attribution model were also evaluated (Supplementary Table S1b) [23]. None of the female MS patients, OIND and control subjects was pregnant before and during the study.

### 2.2. Quantification of Peripheral Blood Antibodies

Anti-HHV antibodies were evaluated in plasma samples from MS and control subjects with diagnostic ELISA kits (EBV, HHV-6, VZV; HSV-1; HSV-2) (Bethyl Laboratories, Montgomery, TX, USA). The endpoint Ig titers were evaluated with serial dilutions (1:10, 1:40; 1:60; 1:110; 1:240; 1:360) of the samples.

### 2.3. Genotyping of KIR and HLA

Genomic DNA was extracted from whole blood (QIAamp DNA Blood Mini kit, Hilden, Germany). The Olerup Typing kit (West Chester, PA, USA) was used to genotype KIR and HLA alleles and to quantify HHVs genomes by real-time PCR (Supplementary Table S2) following the manufacturer’s procedures.

### 2.4. Quantification of Cytokine Levels

The levels of 9 different cytokines (Interferon-gamma (IFN-gamma), Interleukin-1alpha (IL-1alpha), IL-1beta, IL-4, IL-6, IL-8, IL-10, IL12p70, Tumor-necrosis factor alpha (TGF-alpha)) implicated in inflammation control (Aushon, Billerica, MA, USA) were analyzed on serum samples via a Multiplex ELISA kit (Cytokine 2 Array; Chemokine 2 Array). Serum samples were collected in a serum separator tube (SST), spun at 1000 G at room temperature centrifuge for 10–15 min, recovered and stored at −80 °C.

### 2.5. Peripheral Blood Mononuclear Cell (PBMC) Culture

A clinical hematologic analysis showed no differences in cell counts between the three groups (Supplementary Table S1c). PBMCs were extracted from whole blood by Ficoll gradient (Cederlane, Hornby, ON, Canada). After extraction, PBMCs were resuspended in 2 mL of RPMI-1640 (Euroclone, Pavia, Italy) supplemented with 100 U/mL Penicillin G, 2 mM L-Glutamine, 20% FCS and 100 ug/mL Streptomycin (Euro-clone, Pavia, Italy).

Natural killer cells were obtained from peripheral blood samples using the negative magnetic cell separation system (Miltenyi Biotech, Gladbach, Germany) [24]. The NK cell content was >90% as assessed by flow cytometry with CD3-PerCp-Cy5.5, CD56-FITC moAbs staining (e-Bioscience, Frankfurt, DE) (data not shown). NK cells were resuspended at 2 × 10^6^ cells/mL in 20 mL of RPMI 1640 (BioWhittaker) containing 1 mM non-essential amino acids, 10% human AB serum (Mediatech, Herndon, VA, USA), 1 mM pyruvate, 2 mM glutamine, 20 mM HEPES, 100 μg/mL Strep-tomycin and 100 U/mL Penicillin (Gibco BRL Life Technologies). Cells were stimulated with 100 U/mL of IL-2 (Hoffmann-LaRoche) on day 0 and cultured for 5 to 6 days at 37 °C, 5% CO2.

### 2.6. Peripheral Blood Monocyte-Derived Microglia

Monocytes (adherent PBMC) were obtained from PBMCs cultured in T25 tissue culture flasks (2 × 10^6^ to 5 × 10^6^ cells/mL) using RPMI-1640 Glutamax medium (Invitrogen, Milan, Italy) supplemented with 1% antibiotics/antimycotic (10,000 g/mL streptomycin sulfate, 10,000 units/mL penicillin G sodium and 25 g/mL Amphotericin B, Invitrogen). After overnight incubation, adherent cells, which are mainly monocytes (>90%), as confirmed by a flow cytometry analysis, using anti-CD14-FTC and anti-CD16-PE moAbs (BD Biosciences, Milan, Italy), FACSCantoII flow cytometer (BD, Milan, Italy) and FlowJo LLC analysis software (Ashland, Catlettsburg, OR, USA) (Supplementary Figure S1), were used for the generation of microglia (M-MG). The differentiation of PBM-microglia was obtained in 6-well tissue culture plates (Sarstedt, Nümbrecht, Germany) using RPMI-1640 Glutamax supplemented with 1% antibiotic/antimycotic (serum-free condition) and a mixture of human recombinant cytokines, including GM-CSF (10 ng/mL; PeproTech, London, UK), M-CSF (10 ng/mL; PeproTech, London, UK), beta-nerve growth factor (NGF-beta 10 ng/mL; PeproTech, London, UK) and CCL2 (100 ng/mL) for up to 14 days. The generation of M-microglia was confirmed by morphology evaluation and immune-phenotype characterization for the expression of Iba1 by an-ti-Iba-1 PE moAb and of substance P with anti-substance P FITC mouse monoclonal antibody (moAb) [25,26].

### 2.7. HHV Infection

Cell-free virus inocula were obtained as previously described: EBV from lymphoblastoid cell line LCL-B95.8 (kind gift of Professor R. Dolcetti) activated using 20 ng/mL TPA (12-O-tetradecanoylphorbol-13-acetate) (Sigma-Aldrich, St. Louis, MO, USA) [26]; HHV-6A (strain U1102) grown in the J-Jhan cell line (ATCC TIB-153) [27]; HHV-6B (strain Z29) [24] grown in the Sup T1 cell line (ATCC CRL-1942); HSV-1 (strain F) grown on Vero cells (ATCC CCL81); VZV (ATCC VR-1433) grown on MRC5 cells (ATCC CCL171). The infection of M-MG was performed at a multiplicity of infection (m.o.i.) of 0.1 plaque forming unit/cell. A UV-inactivated virus was used as a negative control.

### 2.8. Viral Load Quantification

Overall, 1, 3, 7 and 14 days post-infection, the cells were harvested and DNA was extracted with the GeneAll ExgeneTM Cell SV kit (GeneAll Biotechnology, Seoul, Korea). Real-time PCR for HHVs was performed (as reported in Supplementary Table S2) following the manufacturer’s procedures.

### 2.9. Cytometric Analysis and CD107a Degranulation Assay

NK cells were characterized as CD3 negative cells with a specific CD panel (CD56-FITC, CD16-PerCp-Cy5.5, CD107a-PE) (e-Bioscience, Frankfurt, Germany), anti-KIR2DL2-2DS2-2DL3/CD158b-PE (ThermoScientific, Erembodegem, BE) monoclonal antibodies. Samples were incubated with the moAbs for 30 min in ice and washed. The analysis was performed with FACS CantoII flow cytometer and FlowJo software (Becton Dickinson, San Jose, CA, USA), acquiring 10,000 events. Lymphocytes were identified according to the forward/side scatter profile and NK cells (CD3−/CD56+) were defined and gated within the lymphocyte gate. For the CD107a degranulation assay, after 1 h of incubation at 37 °C and 3 h of treatment with Golgi Stop solution (Becton Dickinson, San Jose, CA, USA), PBMCs were stained. CD158b levels were evaluated in the CD3−/CD56+/CD16+ gated cells. Cell viability was assessed by propidium iodide staining. Anti-isotype controls (Exbio, Praha, Czech Republic) were performed. Ten thousand events were acquired.

### 2.10. Statistical Analyses

The Hardy–Weinberg equilibrium was assessed by the extended version of Fisher’s exact test implemented in Arlequin 3.01. Biological variables were evaluated by Student’s *t* test, Fisher’s exact test and a logistic regression analysis by Graph pad software. Significant *p* values were defined as <0.05. *p* values were corrected (pc) for multiple comparisons, using the Bonferroni correction.

## 3. Results

### 3.1. KIR/HLA Typing

The patients were evaluated for the frequency of KIRs’ receptors and HLA ligands. The results are reported in Table 1. We enrolled 100 MS RR patients, in remission phase (Supplementary Table S1a), 100 healthy controls and 100 subjects with other inflammatory neurological diseases (OIND: neuro-lupus, NLES) (Supplementary Table S1b).

The KIR and HLA allelic distribution was in the Hardy–Weinberg equilibrium. Patients with MS showed a significantly increased frequency of the activating receptor KIR2DS2 (62% vs. 37%; pc: 4.2 × 10^−3^) and a reduced frequency of the activating receptor KIR3DS1 (12% vs. 33%; pc: 4.2 × 10^−3^) in comparison with controls. MS patients had an increased frequency of the inhibitory receptor KIR2DL2 (62% vs. 36%; pc: 3.2 × 10^−3^). No significant differences were found in the distribution of KIR genotypes (AA or Bx) but there was an increase in the HLA-C1/C2 genotype in MS patients (46% vs. 23%; pc: 5 × 10^−3^). The combination KIR2DS2/KIR2DL2 present/C1 present was significantly increased in MS patients in comparison with controls (48% vs. 28%; pc: 0.037) while the combination KIR2DS2/KIR2DL2 absent/C1 present was significantly decreased in MS patients (6% vs. 31%; pc: 5.8 × 10^−5^). The logistic regression analysis showed the combination KIR2DS2/KIR2DL2 present/C1 present strictly correlated with the MS condition (*p* = 0.03; *p* = 0.016, respectively). No significant differences were observed between KIR/HLA frequencies in OIND patients in comparison with controls (Table 1).

### 3.2. Microglial Cells-NK Cells Co-Culture during HHVs’ Infection

**Figure 1:**
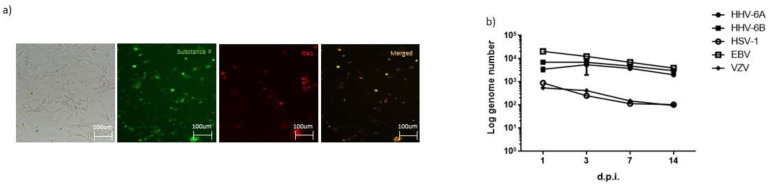
(**a**) Microglial cell obtained from peripheral blood monocytes, stained for substance P and Iba1. (**b**) Viral load in control microglia cells infected with HHVs.

We standardized the protocol to obtain human microglial cells from peripheral blood monocytes. We obtained a sufficient quantity of cells from each sample to carry out the analyses (Figure 1a). The generation of M-MG was confirmed by a morphology evaluation and immune-phenotype characterization for the expression of calcium binding protein Iba1 and substance P, typical characteristics of microglia (Figure 1a). We performed viral infection on microglial cells, obtaining a good viral load after 14 days after infection for HHV-6A, HHV-6B and EBV, while a lower viral replication was observed for HSV-1 and VZV (Figure 1b).

**Figure 2:**
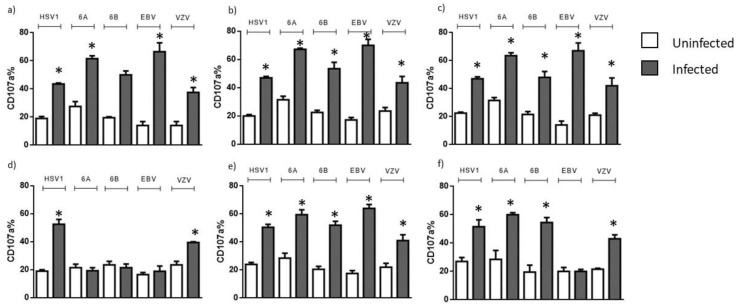
Frequencies of CD107a-positive NK cells after the co-culture with microglia cells infected with HHVs in KIR2DL2-negative (**a**) control, (**b**) MS and (**c**) OIND populations and in KIR2DL2-positive (**d**) control, (**e**) MS and (**f**) OIND populations. * significant *p* value, Student’s *t* test. The data are presented as mean ± SE.

Positive and negative KIR2DL2 natural killer (NK) cells from MS and OIND patients were co-cultured with HSV-1, HHV-6A, HHV-6B, VZV and EBV microglial-infected cells. The activation of NK cells was evaluated as a percentage of NK cells expressing the CD107a degranulation marker. We showed an increase in activated KIR2DL2-negative NK cells from all the three populations in the presence of HSV-1, HHV-6A, HHV-6B, VZV and EBV-infected cells (Figure 2a–c) (*p* < 0.001; t-Student’s test). KIR2DL2-positive NK cells from the control population in the presence of HSV-1, HHV-6A, HHV-6B, VZV and EBV-infected cells showed an increase in CD107a expression (Figure 2d). In contrast, KIR2DL2-positive NK cells from MS patients were not activated in the presence of cells infected with HHV-6A, HHV-6B and EBV (Figure 2e). KIR2DL2-positive NK cells from OIND patients were not activated in the presence of cells infected with EBV (Figure 2f).

**Figure 3:**
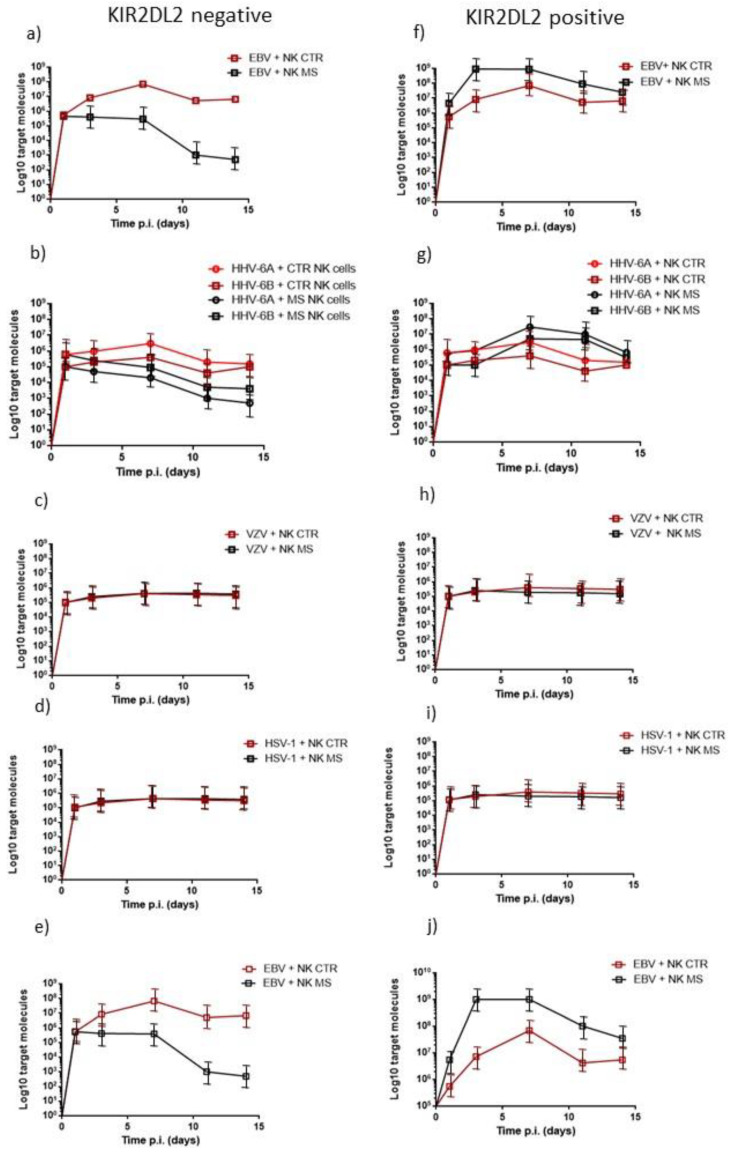
Microglial cells were infected and co-cultured with control or MS NK cells. Viral load was reported in KIR2DL2-negative subjects for (**a**) EBV, (**b**) HHV-6, (**c**) VZV and (**d**) HSV-1. Viral load was reported in KIR2DL2-positive subjects for (**f**) EBV, (**g**) HHV-6, (**h**) VZV and (**i**) HSV-1. Viral load in KIR2DL2-negative OIND patients for (**e**) EBV. Viral load in KIR2DL2-positive OIND patients for (**j**) EBV. NK CTR: NK cells from control subjects; NK MS: NK cells from MS patients. The data are presented as mean ± SE.

On the basis of the results obtained with NK cell activation, we evaluated the viral load in MS patients’ microglia cells. We observed that KIR2DL2-negative subjects from the MS population were able to control viral infections, in agreement with the results obtained from the NK cell activation assay (Figure 3a–d). In particular, KIR2DL2-negative NK cells were able to control EBV, HHV-6A and HHV-6B more efficiently than NK cells from control subjects. KIR2DL2-positive MS patients controlled only VZV and HSV-1 infections similar to control subjects (Figure 3h,i). Interestingly, KIR2DL2-positive OIND patients controlled all the infections apart from EBV infection, similarly to what was observed for NK cell activation (Figure 3j).

**Figure 4:**
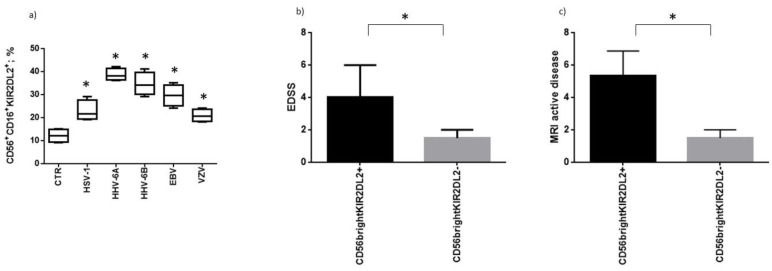
(**a**) Frequency of MS subjects with KIR2DL2 expressing NK cells after the co-culture with microglia cells infected with HHVs. Distribution of MS subjects with KIR2DL2-positive or negative NK cells on the basis of (**b**) EDS and (**c**) MRI disease activity. * significant *p* value, Student’s *t* test. The data are presented as mean ± SE.

When we evaluated the NK cell phenotype from MS patients after the co-culture with microglial-infected cells, we observed a significant increase in KIR2DL2 expression (Figure 4a). The augmented KIR2DL2 expression was more evident in the co-culture with HHV-6A, HHV-6B and EBV-infected microglia cells (Figure 4a). These data are in accordance with the results obtained with viral load quantification and NK cell activation status, supporting the role of KIR2DL2 in controlling NK cell activation in the presence of HHV infections. We evaluated the possible impact of KIR2DL2 expression on clinical status in MS patients. We observed that the subjects that respond with a greater increase of KIR2DL2 expression in the presence of an HHV infection were characterized by a higher EDSS (Figure 4b), supporting an involvement also in disease status. Similarly, the subjects that respond with a greater increase of KIR2DL2 expression in the presence of an HHV infection were characterized by a higher frequency of MRI disease activity (Figure 4c).

### 3.3. Levels of HHVs’ DNA in Peripheral Blood

**Figure 5:**
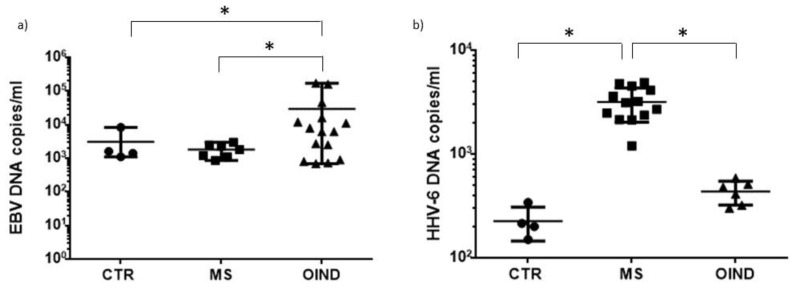
Viral load of (**a**) EBV and (**b**) HHV-6 in control, MS and OIND populations. * significant *p* value, Student’s *t* test. The data are presented as mean ± SE.

The three populations were analyzed for the presence of viral DNA (HSV-1, VZV, EBV, HHV-6) in peripheral blood. The presence of the EBV genome was observed in 10% of healthy controls, 16% of subjects with MS and in 39% of OIND subjects, with a significant difference between MS patients and OIND patients (*p* = 0.0004; Fisher exact test) and OIND patients and controls (*p* < 0.0001; Fisher exact test). Similarly, analyzing the viral load, OIND patients had a higher number of viral genome copies/mL of blood than MS patients (*p* < 0.0001; Student’s *t* test) and controls (*p* = 0.0007; Student’s *t* test) (Figure 5a).

Overall, 10% of controls, 31% of MS patients and 14.6% of OIND patients were positive for the presence of the HHV-6 genome (Figure 5b), with the highest viral load in MS patients in comparison with controls (*p* = 0.001; Student’s *t* test) and OIND patients (*p* < 0.0001; Student’s *t* test). Subjects who tested positive for the presence of the HHV-6 genome in Q-PCR were then characterized for the type of virus, HHV-6A or HHV-6B. The subjects positive for the HHV-6A genome were 60% of MS patients and 30% of OIND patients (*p* < 0.0001; Student’s *t* test), while no control subject was positive for the HHV-6A genome. The subjects positive for the HHV-6B genome were 20% of MS patients, 45% of OIND patients (*p* = 0.0002; Student’s *t* test) and 100% of control subjects (vs. MS and OIND *p* < 0.0001; Student’s *t* test). The subjects positive for HHV-6A/-6B genomes were 20% of MS patients, 25% of OIND patients (*p* = 0.5; Student’s *t* test), while no control subject was positive for HHV-6A/-6B genomes. No samples tested positive for the presence of HSV-1 or VZV DNA as was expected as the site of latency of both these viruses was not the peripheral blood but in ganglion cells. No differences were observed subdividing the samples on the basis of KIR2DL2 positivity (data not shown).

### 3.4. Levels of Antibodies towards HHVs

We evaluated the levels of antibodies (IgM and IgG) towards HHVs (HSV-1, HSV-2; EBV; HHV-6; VZV) in the plasma samples of the three cohorts.

The results obtained by the analysis of EBV EBNA1 IgG evidenced that 75% of MS patients were positive, in comparison with 40% of OIND patients (*p* < 0.0001; Fisher exact test) and 30% of control subjects (*p* < 0.0001; Fisher exact test). By dividing the samples on the presence of the KIR2DL2 receptor, 90% of MS patients were positive for EBV EBNA1 IgG in comparison with 49% of OIND patients (*p* < 0.0001; Fisher exact test) and the 50% of control subjects (*p* < 0.0001; Fisher exact test). The results obtained with EBV VCA IgG were comparable to EBNA1 IgG results.

The results obtained from the VZV IgG analysis showed that 90% of MS and OIND patients and 80% of control subjects were positive for VZV IgG. By dividing the samples according to the presence of the KIR2DL2 receptor, a slight increase in the subjects positive for VZV IgG was observed in OIND subjects (50%) compared to control subjects (35%) (*p* = 0.05; Fisher exact test).

The results obtained from the IgG analysis for HHV-6 showed that 100% of MS patients, 95% of OIND patients and 100% of control subjects were positive for HHV-6 IgG. By dividing the samples on the basis of the presence of the KIR2DL2 receptor, a higher percentage of MS (60%) and OIND (60%) patients tested positive for HHV-6 IgG in comparison with control subjects (40%) (*p* = 0.007, Fisher exact test).

The results obtained from the IgG analysis for HSV-1 showed that 60% of MS patients and 70% of control subjects were positive in comparison with 35% of OIND patients (*p* = 0.007; Fisher exact test). By dividing the samples on the basis of the presence of the KIR2DL2 receptor, a higher percentage of the subjects positive for IgG against HSV-1 was observed in MS patients (60%) and control subjects (65%) in comparison with OIND patients (*p* = 0.0007; Fisher exact test).

**Figure 6:**
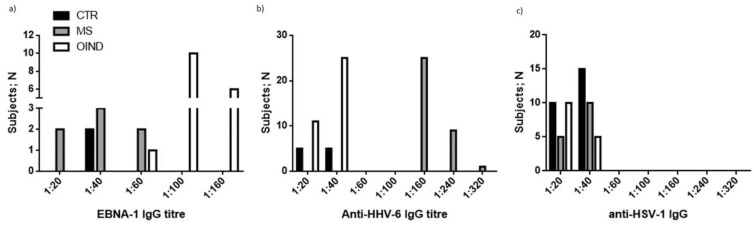
Levels for (**a**) anti-EBNA-1 IgG, (**b**) anti HHV-6 IgG and (**c**) anti HSV-1 IgG in control, MS and OIND populations.

These results showed that the expression of the KIR2DL2 receptor seems to correlate with the increased positivity for anti-EBV, anti-HHV-6 and anti-HSV-1 IgG; we assessed the levels of positivity for EBV, HHV-6 and HSV-1 IgG in the three populations. We performed serial two-fold dilutions of controls and test samples (initial dilution 1:20 *v*/*v*) in dilution buffer (PBS containing 1% skimmed milk). We observed that OIND patients showed the highest titers for EBNA-1 IgG (Figure 6a), while MS patients presented the highest levels for anti-HHV-6 IgG (Figure 6b). The control subjects presented the highest levels for anti-HSV-1 IgG (Figure 6c).

### 3.5. Levels of Inflammatory Cytokines in Plasma Samples

In order to assess the inflammatory state of the subjects, the plasma levels of nine pro- or anti-inflammatory cytokines (IL-1alpha, IL-1beta, INF-gamma, IL-10, IL-4, IL-6, IL-8, IL-12 and TNF-alpha) were evaluated. They were selected as representative of a different immune response and previously implicated in both MS and neuro-lupus diseases [28,29].

**Figure 7:**
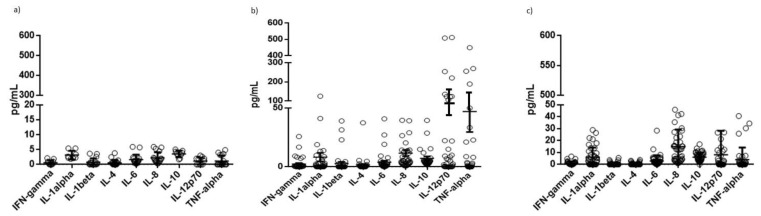
Cytokine serum levels in (**a**) control, (**b**) MS and (**c**) OIND populations. The data are presented as mean ± SE.

MS and OIND patients showed the highest levels of IL-8, IL-12p70, IL-10 and TNF-alpha in comparison with control subjects (Figure 7; *p* <0.0001; Student’s *t* test). Interestingly, MS and OIND patients showed similar levels of IL-8 (*p* = 0.36; Student’s *t* test), while MS patients presented higher IL-12p70, TNF-alpha and IL-10 levels in comparison with OIND patients (*p* < 0.0001; Student’s *t* test). No differences were observed when subdividing the samples on the basis of KIR2DL2 positivity (data not shown).

## 4. Discussion

The results of this study demonstrated that KIR2DL2 expression on NK cells gives MS patients a higher susceptibility to HHV infection, confirming our previous results on HSV-1 [14,15,16]. In particular, KIR2DL2 expression on NK cells reduced NK cell activation and consequently HHVs’ clearance in microglia cells from MS patients. Interestingly, the co-culture of NK cells with HHV-infected microglia cells induced a significant increase in KIR2DL2 expression on CD16+CD56+ NK cells. When we evaluated the control subjects we observed no statistical differences in the activation status of NK cells between KIR2DL2-positive and negative subjects. Interestingly, previous results showed a correlation between KIR2DL2 and the recurrence of HHV infection in healthy subjects [17]. The authors recognized receptor/ligand pair KIR2DL2/HLA-C1 as a predisposing factor for HSV-1 infection and reactivation. These results suggest the implication of KIR2DL2 in HHV infection susceptibility in the human population, that has a deep effect on NK cell activation in MS patients. In particular, we observed that the subjects that respond with a greater increase in KIR2DL2 expression in the presence of an HHV infection were characterized by a higher EDSS, supporting an involvement also in disease status. Similarly, the subjects that responded with a greater increase in KIR2DL2 expression in the presence of an HHV infection were characterized by a higher frequency of MRI disease activity.

We showed an increased susceptibility mainly to EBV and HHV-6 infections in MS patients carrying the KIR2DL2 receptor and HLA-C1 ligand. We can hypothesize that HHV-6 and EBV reactivation, by inducing immune activation via molecular mimicry, may have the ability to induce autoimmunity and cause tissue damage and consequent MS lesion development. HHV-6 and EBV infections are common and have a worldwide distribution, and like most herpesviruses they are a ubiquitous infectious agent, infecting greater than 90% of the world’s population [30,31]. The association between MS and HHV-6 is supported by its ability to productively infect glial cells [32,33,34]; the persistence of HHV-6 DNA for months in the brain [34]; and the high neuropathogenicity of HHV-6 in a marmoset model [35].

As a proof of concept of the increased susceptibility, we evaluated the levels of HHVs’ DNAs. In total, 31% of MS patients, 14.6% of OIND patients and 10% of controls were positive for the presence of the HHV-6 genome, with the highest viral load in MS patients. MS patients presented mainly HHV-6A infection or HHV-6A/-6B co-infection, while OIND patients and controls presented mainly HHV-6B infection. The three cohorts were also evaluated for anti-HHVs IgG. The results obtained by the analysis of EBV EBNA1 IgG evidenced that MS patients presented a higher percentage of positive subjects, mainly in the KIR2DL2-positive group. The results obtained from the IgG analysis for HHV-6 showed a higher percentage of subjects positive for IgG against HHV-6 in KIR2DL2-positive MS and OIND subjects compared to controls. The VZV IgG and HSV-1 IgG analysis showed that MS and OIND patients had the highest percentage of positive subjects, mainly in the KIR2DL2-positive subjects. The increase in only HHV-6 viral load, with the high levels of both EBV and HHV-6 IgG levels in MS patients, sustained a possible increased reactivation of these two viruses in MS patients with low levels of replication but the ability to induce humoral response. This reactivation might result without evident clinical sequelae, but it is able to modify the immune cell status. To evaluate this point, we analyzed the levels of pro- and anti-inflammatory cytokines in serum samples. We observed the highest levels of pro-inflammatory cytokines in MS and OIND [28,29]. In particular, TNF-alpha, IL-8 and IL-12p70 were higher in both MS and OIND patients in comparison with control subjects. These results confirm the enhanced inflammatory status in MS and OIND patients, characterized by an increased cytokine production. Interestingly, there was also an increase in IL-10 levels in MS and OIND patients in comparison with controls, suggesting a role in counteracting the inflammatory envionment in MS and OIND patients.

In fact, EBV reactivation appears to be linked to disease activity in early MS and it has been reported that EBV reactivation in the CNS may play an important role in MS immunopathology [36]. EBNA2 variants have been identified as biomarkers of MS disease course and therapy response [37]. The evaluation of EBV-infected B cells in post-mortem brains of MS cases still provides controversial results [38,39]. However, the efficacy of B cell depleting therapies in relapsing remitting and progressive MS and the pilot trial with in vitro expanded autologous EBV-specific T cell therapy could be considered an indirect evidence of EBV implication in MS, since memory B cells are the target of latent EBV infection [40,41]. Concerning HHV-6, a higher frequency of active infection seems to be related to MS onset [42,43]. In addition, MS plaques showed an increased frequency of HHV-6 DNA and proteins when compared with control tissues [44] and the serum of MS patients presented higher anti-HHV-6 antibody titers than in healthy controls [45]. The possibility to give a complete characterization of NK cell response in each MS patient might help in increasing the safety of therapies with biological and immune-suppressive drugs (e.g., Fingolimod, Natalizumab), that could reactivate HHV infection [46]. Further analyses are needed to confirm these results in a multicentric study, increasing the number of subjects to be enrolled.

## 5. Conclusions

Our results demonstrated that the presence of KIR2DL2 expression on NK cells increased the susceptibility of MS patients to HHV infections. We showed an increased susceptibility mainly to EBV and HHV-6 infections in MS patients carrying the KIR2DL2 receptor and HLA-C1 ligand. The highest HHV-6 viral load was observed in MS patients, with an increased percentage of the subjects positive for IgG against HHV-6 in KIR2DL2-positive MS and OIND subjects compared to controls. MS and OIND patients showed the highest levels of IL-8, IL-12p70, IL-10 and TNF-alpha in comparison with control subjects. Interestingly, MS and OIND patients showed similar levels of IL-8, while MS patients presented higher IL-12p70, TNF-alpha and IL-10 levels in comparison with OIND patients. We can hypothesize that HHVs’ reactivation, by inducing immune activation via molecular mimicry, may have the ability to induce autoimmunity and cause tissue damage and consequent MS lesion development.

The availability of Lirilumab (IPH2102/BMS-986015) [47], a human monoclonal antibody that blocks the interaction of the KIR2DL2 receptor and its ligands, with an ongoing randomized Phase II trial in tumors, could allow a therapeutic approach to promote NK cell activation towards HHV infection in MS patients with a possible rebound in disease outcome. Nevertheless, futher studies are needed to confirm whether these approaches may help in MS clinical management.

## Figures and Tables

**Table 1:** KIR/HLA frequency.

KIR and HLA	MS (*n* = 100)	OIND (*n* = 100)	CNTR (*n* = 100)	p	pc
Activating KIR					
KIR2DS1	50	36	37	0.09	
KIR2DS2	62	34	37	7.0 × 10^−4^	4.2 × 10^−3^
KIR2DS3	36	29	28	0.28	
KIR2DS4	87	84	83	0	
KIR2DS5	45	31	30	0.04	
KIR3DS1	12	32	33	7.0 × 10^−4^	4.2 × 10^−3^
Inhibitory KIR					
KIR2DL1	89	87	86	0.67	
KIR2DL2	62	38	36	4.0 × 10^−4^	3.2 × 10^−3^
KIR2DL3	72	76	77	0.52	
KIR2DL4	98	99	100	0	
KIR2DL5	60	44	46	0.06	
KIR3DL1	94	81	83	0.025	
KIR3DL2	98	99	100	0	
KIR3DL3	98	99	100	0	
KIR genotype					
AA	28	33	35	0.36	
Bx	72	66	65		
HLA genotype					
C1/C1	26	21	20	0.40	
C1/C2	46	25	23	9.9 × 10^−4^	5.0 × 10^−3^
C2/C2	29	44	43	0.055	
HLA-Bw4	78	73	71	0	
HLA-Bw6	92	82	84	0.13	
KIR and their ligands					
KIR2DS1/KIR2DL1 present/HLA-C2 present	40	27	28	0.1	
KIR2DS1/KIR2DL1 present/HLA-C2 absent	10	7	8	0.81	
KIR2DS1/KIR2DL1 absent/HLA-C2 present	2	1	1	0	
KIR2DS2/KIR2DL2 present/HLA-C1 present	48	27	28	0.00561	0.037
KIR2DS2/KIR2DL2 present/HLA-C1 absent	14	14	14	0	
KIR2DS2/KIR2DL2 absent/HLA-C1 present	6	32	31	6.4 × 10^−6^	5.8 × 10^−5^
KIR3DS1/KI3DL1 present/HLA-Bw4 present	30	24	25	0.53	
KIR3DS1/KI3DL1 present/HLA-Bw4 absent	9	6	7	0.80	
KIR3DS1/KI3DL1 absent/HLA-Bw4 present	1	2	3	0.62	

## Data Availability

The data presented in this study are all available upon request.

## Supplementary Materials

The following supporting information can be downloaded at https://www.mdpi.com/article/10.3390/microorganisms10030494/s1: Table S1a: Demographic and clinical characteristics of MS patients; Table S1b: Demographic and clinical characteristics of SLE patients with neuropsychiatric involvement; Table S1c: Clinical hematology results; Table S2: Real-time PCR primers for HHVs’ genome quantitation. Figure S1: Representative staining of monocyte cells obtained from peripheral blood to produce microglia cells. (a) FSC/SSC gating; (b) Monocytes were stained as CD14+CD16−cells.
