# Supplementary material for: Herpesvirus Infections in KIR2DL2-Positive Multiple Sclerosis Patients: Mechanisms Triggering Autoimmunity
Source: Microorganisms. 2022 Feb 23;10(3):494. doi: 10.3390/microorganisms10030494 (PMC8954585; doi:10.3390/microorganisms10030494)
Supplement: Supplementary file 1 [file microorganisms-10-00494-s001.zip › microorganisms-1546395-supplementary.pdf]

microorganisms MDPI

Supplementary Table S1.

(a) Demographic and clinical characteristics of MS patients.

**Female, n (%)**	**52 (52)**
Age (years), mean (SD)	39 (10)
EDSS score, mean (SD)	2.0 (4.0)
Clinical activity (%)	40
MRI activity (%)	35
Frequent herpesvirus reactivations (%)	0 (%)
Neurological manifestation during HHVs primary infection	0 (%)

(b) Demographic and clinical characteristics of SLE patients with neuropsychiatric involvement.

**Female, n (%)**	**54 (54)**
Age (years), mean (SD)	37 (12)
NP, n (%)	
Cerebrovascular disease	15 (37.5)
Seizure disorder	7 (17.5)
Headache	6 (14)
Peripheral neuropathy	4 (10)
Cognitive disorder	2 (5)
Mood disorder	1 (2.5)
Transverse myelitis	1 (2.5)
MS-like syndrome	1 (2.5)
Chorea	1 (2.5)
Acute confusional state	1 (2.5)
Aseptic meningitidis	1 (2.5)
SLEDAI total score, mean (SD)	3.1 (3.5)
SLICC/ACR score (SDI)	1.3 (1.2)
NP score, mean (SD)	6.9 (1.4)
Frequent herpesvirus reactivations (%)	0 (%)
Neurological manifestation during HHVs primary infection	0 (%)

(c) Clinical hematology results. Data are reported as Median values.

**Analyte**	**Result CNTR**	**Result MS patients**	**Result SLE patients**
White blood cells, total	5.60x10^3/µl	5.61x10^3/ µl	5.41x10^3/ µl
RBC	4.60x10^6/ µl	4.62x10^3/ µl	4.52x10^6/ µl
Neutrophils	41.00%	41.30%	40.86%
Lymphocytes	44.70%	44.66%	44.12%
Monocytes	8.60%	8.63%	8.56%
Eosinophils	4.50%	4.48%	4.62%
Basophils	1.20%	1.19%	1.21%

Table S2. Real time PCR primers for HHVs genome quantitation.

**HHVs**	**Microbe Detection Array***
EBV	Vi06439675_s1
HHV-6A/6B	Vi06439627_s1

VZV	Vi06439647_s1
HSV-1	Vi04230116_s1

*Applyed Biosystems Real Time Array.

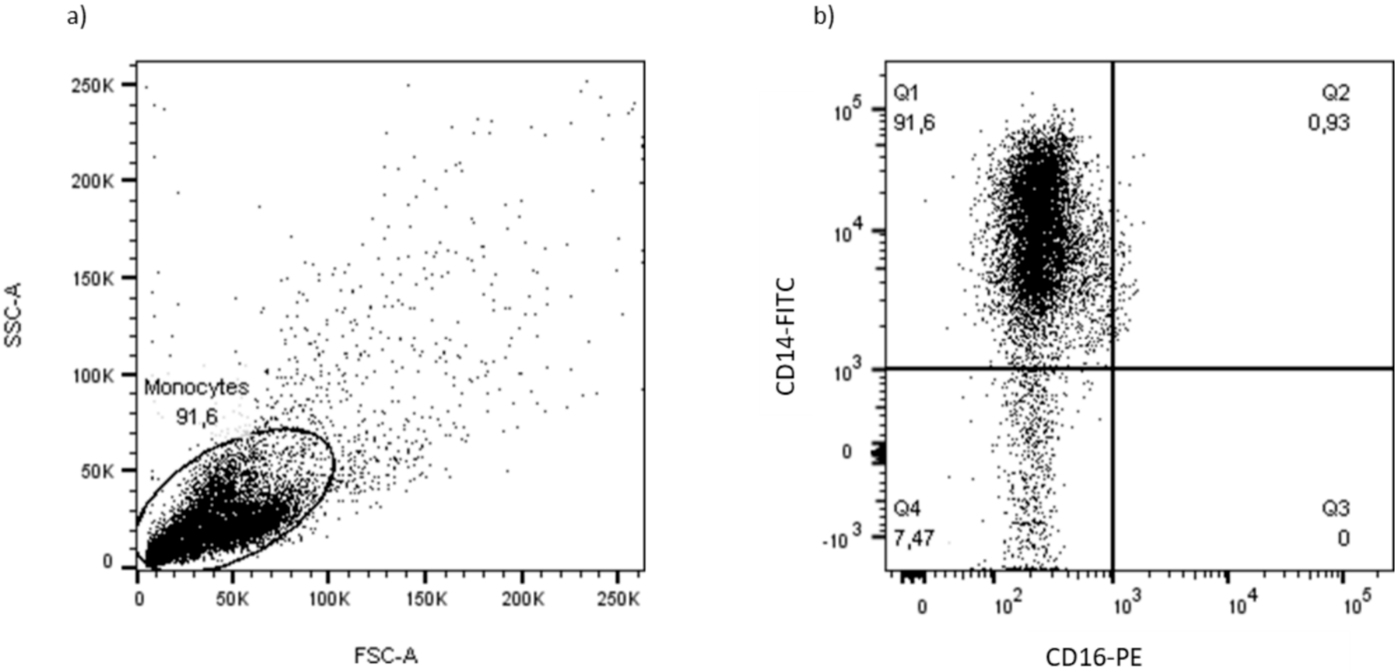

Figure S1. Representative staining of monocyte cells obtained from peripheral blood to produce microglia cells. (**a**) FSC/SSC gating; (**b**) Monocytes were stained as CD14+CD16- cells.
